# Novel Self-Transmissible and Broad-Host-Range Plasmids Exogenously Captured From Anaerobic Granules or Cow Manure

**DOI:** 10.3389/fmicb.2018.02602

**Published:** 2018-11-06

**Authors:** Kosuke Yanagiya, Yoshiaki Maejima, Hiroki Nakata, Maho Tokuda, Ryota Moriuchi, Hideo Dohra, Kengo Inoue, Moriya Ohkuma, Kazuhide Kimbara, Masaki Shintani

**Affiliations:** ^1^Department of Engineering, Graduate School of Integrated Science and Technology, Shizuoka University, Shizuoka, Japan; ^2^Faculty of Engineering, Shizuoka University, Shizuoka, Japan; ^3^Research Institute of Green Science and Technology, Shizuoka University, Shizuoka, Japan; ^4^Department of Agriculture, University of Miyazaki, Miyazaki, Japan; ^5^Japan Collection of Microorganisms, RIKEN BioResource Research Center, Tsukuba, Japan; ^6^Department of Bioscience, Graduate School of Science and Technology, Shizuoka University, Shizuoka, Japan

**Keywords:** plasmid, replication, conjugation, PromA, broad host range

## Abstract

Novel self-transmissible plasmids were exogenously captured from environmental samples by triparental matings with pBBR1MCS-2 as a mobilizable plasmid and *Pseudomonas resinovorans* as a recipient. A total of 272 recipients were successfully obtained as plasmid host candidates from granules of an anaerobic methane fermentation plant and from cow manure. The whole nucleotide sequences of six plasmids were determined, including one IncP-1 plasmid (pSN1104-59), four PromA-like plasmids (pSN1104-11, pSN1104-34, pSN0729-62, and pSN0729-70), and one novel plasmid (pSN1216-29), whose incompatibility group has not been previously identified. No previously known antibiotic resistance genes were found in these plasmids. In-depth phylogenetic analyses showed that the PromA-like plasmids belong to subgroups of PromA (designated as PromAγ and PromAδ) different from previously proposed subgroups PromAα and PromAβ. Twenty-four genes were identified as backbone genes by comparisons with other PromA plasmids. The nucleotide sequences of pSN1216-29 share high identity with those found in clinical isolates. A minireplicon of pSN1216-29 was successfully constructed from *repA* encoding a replication initiation protein and *oriV*. All the captured plasmids were found to have a broad host range and could be transferred to and replicated in different classes of *Proteobacteria*. Notably, *repA* and *oriV* of pSN1216-29 showed high similarity with one of two replication systems of pSRC119-A/C, known as a plasmid with multidrug resistance genes found in *Salmonella enterica* serovar Senftenberg. Our findings suggest that these “cryptic” but broad-host-range plasmids may be important for spreading several genes as “vehicles” in a wider range of bacteria in natural environments.

## Introduction

Conjugative plasmids are major mobile genetic elements transmitting various accessory genes that confer antibiotic and/or heavy-metal resistance, pathogenicity, and metabolic pathways onto their hosts (Hulter et al., 2017). Identification of plasmids that are conjugative in environmental samples is important not only for understanding the mechanisms of bacterial evolution and adaptation but also for preventing unintentional spread of plasmids with antibiotic resistance genes. Recently, the whole nucleotide sequences of >10,000 plasmids became available in a public database^1^, but these data do not necessarily tell us whether they can spread among different bacteria. Collection of plasmids from environmental samples on the basis of their conjugative functions would therefore help to understand which plasmids can be transferred in nature. Exogenous plasmid capture techniques have been applied to obtain a variety of plasmids from activated sludge, manure, and a rhizosphere (Smalla et al., 2015). Among them, triparental mating using a donor with a mobilizable plasmid is an efficient method for collecting conjugative plasmids in environments without any marker genes, including antibiotic or heavy-metal resistance genes or metabolic genes (Smalla et al., 2015). This method is dependent on the mobilizing ability of a self-transmissible plasmid from environmental samples, which can act as a “helper” plasmid for a previously known mobilizable plasmid (Hill et al., 1992; Top et al., 1994). One study revealed successful isolation of new broad-host-range plasmids from freshwater by the triparental mating methods (Brown et al., 2013). Using derivatives of pBBR1MCS (Kovach et al., 1994)—whose Inc group has not been identified—as a mobilizable plasmid, those authors isolated a new plasmid, pMBUI2, in addition to the IncP-1, IncU, and IncN plasmids from freshwater samples (Brown et al., 2013). This finding implies that the triparental method involving pBBR1MCS vectors is appropriate for collecting new types of self-transmissible plasmids from environmental samples.

In the present study, we isolated and characterized new conjugative plasmids from granules collected at an anaerobic waste water treatment plant and from cow manure collected in Japan.

## Materials and Methods

### Bacterial Strains, Plasmids, and Culture Conditions

The bacterial strains and plasmids used in this study are listed in Table 1. *Ensifer, Delftia, Hydrogenophaga, Ochrobactrum, Pseudomonas*, and *Rhizobium* strains were cultivated in Luria broth (LB) (Sambrook and Russell, 2001) at 30°C, and *Escherichia coli* JM109 and S17-1λ*pir* (Simon et al., 1983)—for construction of derivative strains—were grown in LB at 37°C. R2A plates containing 1.5% agar were employed for filter matings. Ampicillin (Ap, 50 μg/mL), chloramphenicol (Cm, 30 μg/mL), kanamycin (Km, 30 μg/mL for capturing and 50 μg/mL for other purposes), gentamicin (Gm, 30 μg/mL), rifampicin (Rif, 30 μg/mL for capturing and 50 μg/mL for other purposes), and tetracycline (Tc, 12.5 μg/mL for *E. coli* and 50 μg/mL for the other microbes) were added to the medium. Cycloheximide (100 μg/mL) was added to prevent the growth of fungi. For plate cultures, LB was solidified by means of 1.5% agar (w/v).

**Table 1:** Bacterial strains and plasmids used in this study.

Strain or plasmid	Relevant characteristics	Reference
**Bacterial strains**		
*Delftia acidovorans* JCM 5833^T^	*Betaproteobacteria*, type strain, obligate aerobe	BRC-JCM, RIKEN
*Ensifer terangae* JCM 20965	*Alphaproteobacteria*, facultative anaerobe	BRC-JCM, RIKEN
*Escherichia coli*		
JM109	F′ [*tra*D36, *pro*AB, *lacI*^q^, *lac*ZΔM15], *rec*A1, *end*A1, *gyr*A96, *thi-1, hsd*R17(r_K_^-^ m_K_^+^), e14^-^ (*mcr*A^-^), *sup*E44, *rel*A1, Δ(*lac*-*pro*AB)	RBC Bioscience
S17-1λ*pir*	Tm^r^, Sm^r^, *recA, thi, pro, hsdR^-^M*^+^, RP4: 2-Tc:Mu: Km Tn*7* *λpir*	Simon et al., 1983
*Hydrogenophaga pseudoflava* JCM 21410^T^	*Betaproteobacteria*, facultative anaerobe	BRC-JCM, RIKEN
***Pseudomonas putida***		
KT2440RG	Derivative strain of KT2440, spontaneously Rif^r^ with Gm^r^ gene into chromosome, obligate aerobe	Shintani et al., 2005
SMDBS	SM1443 (derivative strain of KT2440), *lacI*^q^ gene in its chromosome, and *dapB* was deleted.	Shintani et al., 2014
SMDBS(pSN1104-11, pBBR1MCS-2)	SMDBS bearing pSN1104-11 and pBBR1MCS-2 (Km^r^)	This study
SMDBS (pSN0729-62, pBBR1MCS-2)	SMDBS bearing pSN0729-62 and pBBR1MCS-2 (Km^r^)	This study
SMDBS(pSN1216-29, pBBR1MCS-2)	SMDBS bearing pSN1216-29 and pBBR1MCS-2 (Km^r^)	This study
SMDBS(pSN1104-11, pBBR1MCS-5)	SMDBS bearing pSN1104-11 and pBBR1MCS-5 (Gm^r^)	This study
SMDBS(pSN0729-62, pBBR1MCS-5)	SMDBS bearing pSN0729-62 and pBBR1MCS-5 (Gm^r^)	This study
SMDBS(pSN1216-29, pBBR1MCS-5)	SMDBS bearing pSN1216-29 and pBBR1MCS-5 (Gm^r^)	This study
***Pseudomonas resinovorans***		
CA10dm4R	Derivative strain of CA10dm4 spontaneously Rif^r^.	Shintani et al., 2005
CA10dm4RGFP	CA10dm4R, miniTn*7*(Gm) P_A1/O4/O3_-*gfp*-a was inserted into chromosome (Gm^r^, Cm^r^).	This study
*Rhizobium soli* JCM 14591^T^	*Alphaproteobacteria*, type strain, facultative anaerobe	BRC-JCM
**Plasmids**		
pBBR1MCS-2	Km^r^, *lacZα* *mob*; compatible with IncP, IncQ, and IncW plasmids	Kovach et al., 1995
pBBR1MCS-3	Tc^r^, *lacZα* *mob*; compatible with IncP, IncQ, and IncW plasmids	Kovach et al., 1995
pBBR1MCS-5	Gm^r^, *lacZα* *mob*; compatible with IncP, IncQ, and IncW plasmids	Kovach et al., 1995
pSN1216-29ori001	1640-bp DNA region containing *repA*, three DnaA boxes, four iterons, and AT-rich region of pSN1216-29 connected with Tc^r^ gene of pBBR1MCS-3	This study
pSN1216-29ori002	1590-bp DNA region containing *repA*, three DnaA boxes and four iterons of pSN1216-29 connected with Tc^r^ gene of pBBR1MCS-3	This study
pSN1216-29ori003	1491-bp DNA region containing *repA*, two DnaA boxes and two iterons of pSN1216-29 connected with Tc^r^ gene of pBBR1MCS-3	This study
pSN1216-29ori004	1350-bp DNA region containing *repA* connected with Tc^r^ gene of pBBR1MCS-3	This study

### Exogenous Plasmid Capture

Triparental exogenous isolation of plasmids was performed via a donor strain of *E. coli* with pBBR1MCS-2 (Kovach et al., 1995) and a GFP (green fluorescent protein)-tagged recipient, *P. resinovorans* CA10dm4RGFP (methods for preparation of the recipient strain are described in Supplementary Text S1). The granules were sampled from a lab scale upflow anaerobic sludge blanket (UASB) reactor for methane fermentation (total volume was 1 L) on September 11, 2015; November 4, 2015; December 7, 2015; and May 17, 2016. The reactor was supplied with 0.3 g/L glucose, 1.45 g/L K_2_HPO_4_, and 0.75 g/L KH_2_PO_4_ as model waste water, and the other conditions were implemented similarly as described elsewhere (Suzuki et al., 2015). The cow manure was sampled from cows that were not fed with antibiotics, in the Sumiyoshi field of the University of Miyazaki, Japan, on April 11, 2016; October 11, 2016; and May 16, 2017. Then, 1 g (wet weight) of each sample potentially containing helper bacterial cells with self-transmissible plasmids was resuspended in 10 mL of PBS. Large particles were precipitated after incubation of the samples for up to 30 min at room temperature, and then the supernatants (5 mL for granules and 500 μL for cow manure) were used for subsequent experiments. The overnight-cultured donor and recipient strains were mixed with the above-mentioned environmental samples with helper strains on a membrane filter (0.2 μm pore size; Advantec, Dublin, CA, United States) on LB containing cycloheximide for 48 h at 30°C (filter mating). After that, the mixture on the filter was collected and resuspended in 5 mL of PBS, and then 100 μL of a serial dilution was spread on LB with Rif, Km, and Gm. The colonies with green fluorescence were isolated and then subjected to the following genetic analyses.

### DNA Manipulations

Total DNA from bacterial strains was extracted by using the NucleoSpin^®^ Tissue Kit (TAKARA BIO). Total DNA from isolates for PCR was extracted and purified from isolates by means of an AcroPrep^TM^ Advance 96 Filter Plate (Pall Life Sciences, Westborough, MA, United States) after lysis of 10 μL of the cultured isolate with 0.5% sodium dodecyl sulfate (SDS) and 0.1 μg/μL proteinase K. Small plasmids were extracted from *E. coli* by the alkaline lysis method (Sambrook and Russell, 2001) or by using the NucleoSpin^®^ Plasmid EasyPure Kit (Takara Bio, Shiga, Japan). Confirmation of the presence of environmental plasmids obtained by the exogenous plasmid capture method in the recipient cells was performed by alkaline lysis extraction and agarose gel electrophoresis. The alkaline lysis extraction was carried out as previously described (Sobecky et al., 1998) with the following modifications: 1 mL of an overnight cell culture in LB was resuspended in 250 μL of solution A (2 mg of lysozyme per milliliter, 0.1 M glucose, 25 mM Tris-HCl [pH 8.0], 25 mM EDTA) and incubated at 37°C for 30 min. Then, 125 μL of solution B (0.3 M NaOH, 2% SDS) was added, and the suspension was mixed by inversion of the tube several times and incubated on ice for 20 min. After that, 180 μL of 3 M sodium acetate (pH 4.8) was added, and the suspension was mixed gently and incubated at room temperature for 10 min. The resultant sample was centrifuged (15,000 ×*g*, 15 min, 4°C), and then the supernatant (∼200 μL) was carefully transferred to a new 1.5 mL tube and was extracted with 100 μL of phenol–chloroform–isoamyl alcohol (25:24:1), the phases were separated by centrifugation (15,000 ×*g*, 15 min), and the aqueous layer was subjected to agarose gel electrophoresis. After confirmation of the presence of plasmids, environmental plasmids for next-generation sequencing (NGS) were extracted by using the Large Construct Kit (Qiagen, Hilden, Germany).

Polymerase chain reaction (PCR) was carried out on a T100^TM^ thermal cycler (Bio-Rad, Hercules, CA, United States) with TaKaRa Ex Taq^®^ (Takara Bio) and the primer set for PromA plasmids, or PrimeSTAR^®^ GXL (Takara Bio) for other plasmids. All primers are listed in Supplementary Table S1. The amplification conditions were as follows: initial denaturation at 96°C for 3 min, followed by 35 cycles of 96°C for 45 s, 57°C for 1 min, and 72°C for 1 min; and then the final extension at 72°C for 7 min with TaKaRa Ex Taq^®^ (for PromA); or 30 cycles of 98°C for 10 s, 55°C for 15 s, and 68°C for 1 min with PrimeSTAR^®^ GXL (for the other plasmids). Restriction enzymes (New England Biolabs or Takara Bio), the HiYield^TM^ Gel/PCR DNA fragments Extraction kit (RBC Bioscience, New Taipei City, Taiwan), a Gibson Assembly system or NEBuilder Hifi DNA Assembly system (New England Biolabs, Ipswich, MA, United States), and competent *E. coli* JM109 cells (RBC Bioscience) were employed for cloning of DNA fragments. All the other procedures were performed according to standard methods (Sambrook and Russell, 2001).

### Sequencing and Annotation

The nucleotide sequences of plasmid DNAs were determined on the MiSeq platform (Illumina, San Diego, CA, United States). Detailed information about plasmid DNA sequences and analyses is given in Supplementary Table S2. Plasmid DNAs were fragmented using the Covaris Acoustic Solubilizer (Covaris, Woburn, MA, United States), and then paired-end libraries were prepared with the TruSeq DNA PCR-Free Library Prep Kit or TruSeq Nano DNA Library Prep Kit (Illumina). Raw (251 or 301 bp paired-end) sequence reads were filtered in the Trimmomatic software (Bolger et al., 2014) by trimming adapter sequences, low-quality ends (quality score, <15), the last 251 or 301 bases, and reads less than 150 bp. The khmer software (Crusoe et al., 2015) served to filter reads with low *k*-mer coverage (<4 or 5) to remove sequences contaminated by the host bacterial genome. The high-quality reads were assembled in the SPAdes software (Bankevich et al., 2012) with a default set of *k*-mer sizes, and the resultant contigs were manually closed by removal of 127 bp overlapping ends. The finished sequences were confirmed by mapping the high-quality reads in BWA-MEM and were visualized in Integrative Genomics Viewer (Thorvaldsdottir et al., 2013).

The first annotations were performed by means of DFAST (Tanizawa et al., 2018) and then corrected manually. The annotated genes in pSN1104-59 were reannotated and named based on those in R751 (Thorsted et al., 1998), except for *kfrB* (*upf54.8* in R751) and *kfrC* (*upf54.4* in R751). Similarly, those in pSN1104-11, pSN1104-34, pSN0729-62, pSN0729-70, and pSN1216-29 were annotated on the basis of other similar plasmids, and several genes putatively involved in conjugation were renamed as described elsewhere (Thomas et al., 2017).

### Bioinformatic Analyses

The nucleotide sequences of genes encoding a replication initiation protein (*trfA* or *repA*) were aligned in ClustalW (Thompson et al., 1994), and the maximum likelihood method was used for the unrooted trees in MEGA 7 (Kumar et al., 2016). Comparative analyses between the plasmids obtained in the present study and other previously known plasmids were performed and visualized in Easyfig ver. 2.2.2 (Sullivan et al., 2011). Visualization of plasmid maps was performed using SnapGene^2^. Identification of core gene sets of PromA plasmids was performed in Easyfig, and pairwise distances among core gene products were calculated in MEGA 7 by the Jones–Taylor–Thornton method (Norberg et al., 2011). GC content and codon usage of plasmids were analyzed via the G-language System (Arakawa et al., 2008).

### Transferability of Plasmids

Transferability of the obtained plasmids (pSN1104-11, pSN1104-34, pSN0729-62, pSN0729-70, and pSN1216-29) was confirmed by filter mating assays with *Pseudomonas putida* SMDBS as a donor of each plasmid with mobilizable plasmids pBBR1MCS-2 or pBBR1MCS-5 (see Supplementary Text S1). As for recipients, bacterial strains belonging to different classes of *Proteobacteria* were used, including *Ensifer terangae* JCM 20965, *Ochrobactrum anthropic* JCM 21032^T^, and/or *Rhizobium soli* JCM 14591^T^ as *Alphaproteobacteria* as well as *Delftia acidovorans* JCM 5833^T^ and/or *Hydrogenophaga* *pseudoflava* JCM 21410^T^ as *Betaproteobacteria* (see Supplementary Text S1). After transconjugant candidates were obtained, the presence of each plasmid was confirmed by PCR with *repA-*specific primers (Supplementary Table S1). To test whether they were derivatives of recipients, repetitive extragenic palindromic PCR (BOX-PCR) was conducted with the BOXA1R primer (5′-CTACGGCAAGGCGACGCTGACG-3′) (Versalovic et al., 1994; Shintani et al., 2011).

### Identification of a Minimum DNA Region of pSN1216-29 for Its Replication

Putative promoter sequences of the *repA* gene in pSN1216-29 were predicted in BPROM (Solovyev, 2011). The DNA region containing *repA* and different fragments of the *oriV* region (1640, 1590, 1491, and 1350 bp) or the Tc resistance gene were amplified by PCR with primers listed in Supplementary Table S1, and pSN1216-29 or pBBR1MCS-3 served as a template (see Supplementary Text S1 for details). The resultant fragments (one of the four above-mentioned amplicons and a fragment with the Tc^r^ gene) were connected by using the NEBuilder HiFi DNA Assembly Master Mix (New England Biotech), yielding pSN1216-29ori001 to pSN1216-29ori004 (Table 1). Transformation of *E. coli* JM109 with each of the resultant plasmids (at most 0.01–0.02 pmol) was performed, and the suspension was spread on LB + Tc plates. Colonies were isolated after an overnight incubation at 37°C, and then genetic analyses were performed by extraction of plasmids to confirm their nucleotide sequences.

### Accession Numbers of Nucleotide Sequence Data

The nucleotide sequence data on the plasmids were deposited in the DDBJ/EMBL/GenBank under accession numbers AP018705 (pSN0729-62), AP018706 (pSN0729-70), AP018707 (pSN1104-11), AP018708 (pSN1104-34), AP018709 (pSN1104-59), and AP018710 (pSN1216-29).

## Results and Discussion

### Classification of the Obtained Transconjugants

A total of 272 GFP-positive colonies were successfully obtained from granule and cow manure samples in exogenous plasmid capturing experiments (Supplementary Table S3). The isolates with putative environmental plasmids were classified by PCR with the specific primer sets to detect different Inc groups including IncA and IncC (Carattoli et al., 2005), IncW (Carattoli et al., 2005), IncP-1 (subgroups αβ𝜀, δ, and γ) (Bahl et al., 2009), and PromA (Zhang et al., 2015). PCR products were obtained with IncP-1– and PromA-specific primers for four and 90 isolates (“IncP-1–positive” or “PromA-positive,” whereas both products were obtained from two isolates), respectively, whereas 180 isolates yielded no amplicons with any primer set (“PCR-negative,” Supplementary Table S3). It is therefore possible that these isolates contain novel plasmids that were undetectable by PCR with the above primers; however, the presence of the plasmids was confirmed in only 125 isolates (Supplementary Table S3). Several plasmids might be unstable and lost from the recipient cells. Among the 125 isolates, six (one IncP-1[αβ𝜀]–positive, two PromA-positive, and three PCR-negative) were chosen from granule and manure samples for whole-plasmid sequencing.

**FIGURE 1:**
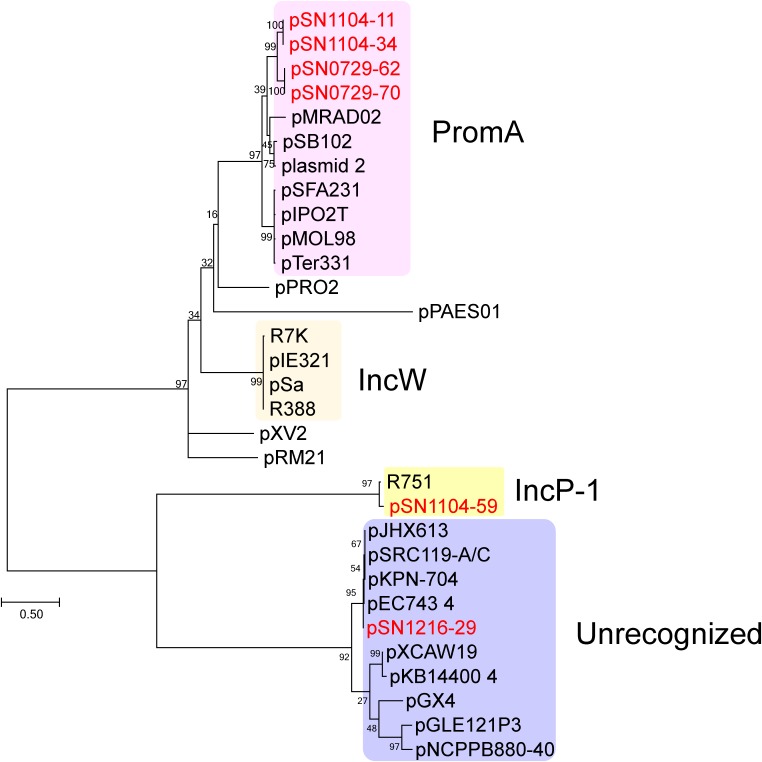
Phylogenetic analyses of captured plasmids and other related plasmids. A phylogenetic tree was constructed from amino acid sequences of a replication initiation protein (TrfA for IncP-1 plasmid, Rep or RepA for the others) by the maximum likelihood method with bootstrap percentages indicated at the nodes (Tamura-Nei model). A scale bar (0.50) shows substitutions per amino acid position. Previously known Inc groups are highlighted in colors, and a previously unrecognized plasmid group contains pSN1216-29. The accession numbers of the references are as follows: pPRO2 (CP000484), R7K (AM901564), pIE321 (EF633507), pSa (U30471), R388 (NC_028464), pXV2 (AF201825), pRM21 (U10426), pXCAW19 (NZ_CP009038), pKB14400_4 (CP014678), pGX4 (NZ_CP004364), pGLE121P3 (KC542383), and pNCPPB880-40 (JQ418534). Other references of PromA, IncP-1, and previously unrecognized plasmid groups are listed in Supplementary Table S5.

The sizes and GC content of the plasmids according to their whole sequences are detailed in Table 2, and their coding sequences (CDSs) are listed in Supplementary Table S4. No antibiotic resistance genes were found in these plasmids. For the first step of the classification, amino acid sequences of putative replication initiation proteins were phylogenetically analyzed along with homologous protein sequences found by BLAST (Figure 1). Two PCR-negative plasmids, pSN0729-62 and pSN0729-70, were found to contain a *repA* product sequence that is similar to that of the PromA-positive plasmids pSN1104-11 and pSN1104-34. The other PCR-negative plasmid, pSN1216-29, contains a *repA* gene that shares high identity with that of several plasmids whose Inc group was not yet identified (Figure 1). Hereafter, the features of these plasmids are described according to this grouping.

**Table 2:** Sequenced plasmids in this study.

Plasmid name	Source	Inc group	Size (bp)	G + C content (%)
pSN1104-11	Granule	PromAγ	41,033	63.69
pSN1104-34	Granule	PromAγ	41,117	63.71
pSN1104-59	Granule	IncP-1β	50,476	65.47
pSN1216-29	Cow manure	new group	35,552	61.76
pSN0729-62	Cow manure	PromAδ	38,644	54.19
pSN0729-70	Cow manure	PromAδ	39,117	54.30

### The pSN1104-59 Plasmid Is an IncP-1β Plasmid

The IncP-1 (IncP-1αβ𝜀)-positive plasmid, pSN1104-59, turned out to be 50,476 bp long. On the basis of the nucleotide sequences of previously known IncP-1 plasmids, phylogenetic analyses were conducted with the nucleotide sequences of genes *trfA* and *traI* of representative IncP-1 plasmids. As presented in Supplementary Figure S1, these genes of pSN1104-59 ended up in the same clades with those of other IncP-1β-1 plasmids (Sen et al., 2013), indicating that they belong to IncP-1β-1. It was found to contain a transposon with a *tnpA* gene encoding a transposase, 33 bp inverted repeats (orf30-*tnpA*) (Supplementary Figure S2 and Supplementary Table S4-1), and 5 bp direct repeats (5′-AGGGC-3′), parts of which were highly conserved in pESA2 (>99% at the nucleotide sequence level, found in *Cronobacter sakazakii*, accession No. CP000784) and/or pKPN_CZ found in *Klebsiella pneumoniae* (Dolejska et al., 2013). The element contained CDSs encoding two putative recombinases, one cation transporter, and five hypothetical proteins (Supplementary Table S4-1). The insertion site of this element was located between genes *upf30.5* and *trbP* (Supplementary Figure S2), which is known as a hot spot for insertions of accessory genes in IncP-1 plasmids (Sota et al., 2007; Norberg et al., 2011).

### Plasmids pSN1104-11, pSN1104-34, pSN0729-62, and pSN0729-70 Are Members of New Subgroups of PromA

Each of the four plasmids, pSN1104-11, pSN1104-34, pSN0729-62, and pSN0729-70, was found to contain a *repA* gene whose products showed high identity with those of the PromA plasmids, which were previously proposed to be a new group of broad-host-range plasmids (Van der Auwera et al., 2009). To date, six plasmids have been reported with their whole nucleotide sequences, including pMRAD02 (Ito and Iizuka, 1971; Van der Auwera et al., 2009), pSB102 (Schneiker et al., 2001), pMOL98 (Top et al., 1994; Gstalder et al., 2003; Van der Auwera et al., 2009), pIPO2T (Tauch et al., 2002), pTer331 (Mela et al., 2008), and pSFA231 (Li et al., 2014). In addition, the nucleotide sequences of *repA* genes in the obtained plasmids share high identity with those in two other plasmids, pXI1 (accession No. NZ_CP020047) in *Thiomonas intermedia* ATCC 15466, a facultative autotrophic sulfide-oxidizing bacterium, and “plasmid 2” in one of the clinical isolates of *Burkholderia pseudomallei*, strain TSV202 (accession No. NZ_CP009154) (Johnson et al., 2015) (Supplementary Table S5). Considering that RepA of PromA plasmids showed identity with those in the IncW plasmids (Gstalder et al., 2003), comparisons with several IncW plasmids were also performed, but the four obtained plasmids ended up in distinct clades in the phylogenetic trees, suggesting that the four obtained plasmids are members of the PromA plasmid family (Supplementary Figure S3). Among the PromA plasmids, RepAs of pSN1104-11 and pSN1104-34 and those of pSN0729-62 and pSN0729-70 were identical because they were found to contain nearly identical *repA* genes (the former two plasmids showed 100% identity, and the latter two 99.6%: a 1264/1269 match). Notably, RepA sequences of these four plasmids were in different clades and were phylogenetically distant from those of the other PromA plasmids (Supplementary Figure S3).

**FIGURE 2:**
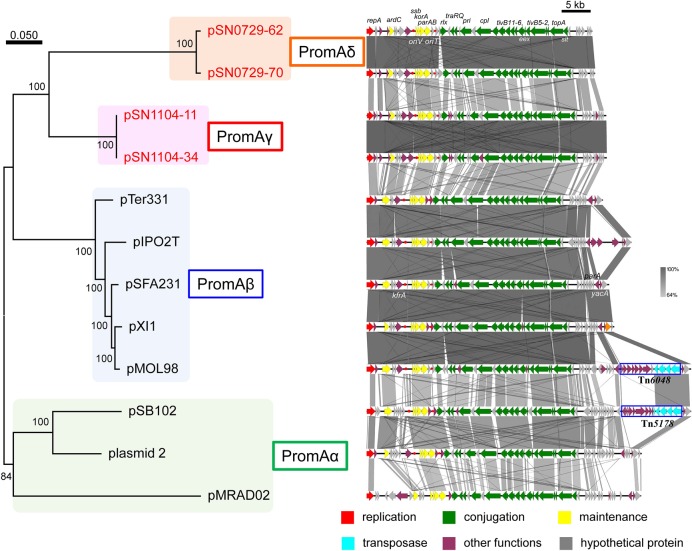
Phylogenetic analyses of 24 concatenated genes conserved in other PromA group plasmids **(left)**, and alignment of 12 plasmids **(right)**. **(Left)** A phylogenetic tree was constructed from nucleotide sequences of 24 core genes by the maximum likelihood method with bootstrap percentages indicated at nodes (Tamura–Nei model). A scale bar (0.050) indicates substitutions per nucleotide position. **(Right)** Coding sequences of each plasmid are presented as colored arrows in accordance with their putative functions. Sequences of *oriV* and putative *oriT* are indicated in red circles. Accession numbers of similar plasmids are shown in Supplementary Table S5.

Another research group compared the whole sequences of PromA plasmids and proposed a common backbone for them (Li et al., 2014). Comparisons of the structures of the PromA plasmids including our four plasmids were carried out based on BLASTN analyses. In the comparisons with the report by Li et al. (2014), the “replication” (*rep, oriV*) and most of the “conjugation” regions (*traA* to *traS*, renamed as *rlx, pri, cpl, tivB11-6, eex, tivB5-2, topA* *slt*, and *trbA*; see below and Supplementary Figure S4) were highly conserved, but only a part of the region for “maintenance/control” was found to be conserved (i.e., *ardC, korB/parB, incC/parA, korA*, and *ssb* are conserved, whereas *yacA, parA*, or *kfrA* are not; Figure 2). These 24 genes were designated as core genes for the PromA plasmids. As for the regions for conjugation, several genes did not match one another; for example, putative relaxase genes had the name as *traS* in pSFA231, pMOL98, pSB102, and pMRAD02 but as *traR* in pTer331 and pIPO2. Likewise, the corresponding genes of *traQ* and *traR* in pSFA231 were named as *traS* and *traQ* in pTer331 and pIPO2T. Next, similar genes found in the four obtained plasmids were renamed according to a recently published review (Thomas et al., 2017) (Supplementary Figure S4 and Supplementary Tables S4-2,S4-5). The MOB type and MPF class were MOB_P_ and MPF_T_, respectively, according to a classification proposed previously (Smillie et al., 2010). Overall, 24 conserved genes in all 12 plasmids were found to be backbone genes (core genes) for these PromA plasmids (Figure 2 and Supplementary Table S6). Pairwise distances among all their products were calculated (Supplementary Table S6), and phylogenetic analysis was performed with the concatenated nucleotide sequences of the 24 genes. These genes are completely conserved between pSN1104-11 and pSN1104-34 (Supplementary Table S6). In these comparisons, four plasmids ended up in two distant clades, both of which were different from those of the PromAα and PromAβ subgroups, suggesting that these could be designated as subgroups PromAγ and PromAδ (Figure 2). The *oriV* region of the PromA plasmid was previously experimentally identified in pMOL98 (Gstalder et al., 2003), and was previously predicted to be the *oriV* region of pSFA231 according to the sequences of pMOL98 (Li et al., 2014). Although the *ardC*-*kfrA* region was found to be not very conserved among several PromA plasmids, the upstream sequence of *ssb* showed relatively high identity (Figure 2), indicating the presence of an *oriV* region in the PromA plasmids.

### Plasmids pSN0729-62 and pSN0729-70 Have Lower G + C Content Than Do the Other PromA Plasmids

Of note, GC content of PromAδ plasmids, pSN0729-62 and pSN0729-70, turned out to be lower than that of the other PromA plasmids, and the PCR products were not produced by PromA-specific primers (repA-1 and repA-2, Supplementary Table S1). GC content among pSN1104-11 (63.7%), pSN1104-34 (63.7%), pSN0729-62 (54.2%), and pSN0729-70 (54.3%) is within a 10% range (Table 2). Moreover, every core gene of PromAδ plasmids showed uniformly lower GC content than that of the other PromA plasmids (Supplementary Table S7). Forty genes (more than 74% of annotated genes in the plasmids) were found to be conserved in subgroups PromAγ and PromAδ (Supplementary Table S8), suggesting that these two subgroups are very closely related to each other. GC contents of these conserved genes showed ∼10% differences (65% for PromAγ and 55% for PromAδ on average), and the third positions of codons had ∼20% differences (82% for PromAγ and 61% for PromAδ on average; Supplementary Table S8). Considering the differences in the number of CDSs in PromAγ and PromAδ plasmids, the numbers of amino acids of the products of genes in each plasmid were similar (Supplementary Table S9-1). Notably, PromAδ plasmids contain more AT-rich codons than do PromAγ plasmids (Supplementary Table S9-2). Thus, one of the reasons for the changes in GC content of these two PromA subgroups was probably changes in codon usage, which might have occurred in their different hosts for adaptation to their host environments. Indeed, it was revealed elsewhere that GC content is lower in plasmids than in host chromosomes (Rocha and Danchin, 2002; van Passel et al., 2006), and that there is a strong correlation in terms of GC content between plasmids and host chromosomes (Nishida, 2012). Therefore, the difference in GC content between the two subgroups of the isolated PromA plasmids could be associated with changes in their host ranges. Obviously, these two plasmids can be transferred among *Gammaproteobacteria* strains because they were successfully transferred from their original hosts (in each environmental sample) to *E. coli* with pBBR1MCS-3, and then transferred to *P. resinovorans* during the exogenous capture. Similarly, the plasmids were successfully transferred to *P. putida*. Moreover, transconjugants of *Betaproteobacteria* with pSN1104-11 or pSN0729-62 could not be detected; however, the previous hosts of PromA plasmids (pTer331, pXl1, plasmid_2, pMRAD020) belong to *Betaproteobacteria* (Supplementary Table S5). Of note, pSN1104-11 was capable of transfer to *O. anthropi* but not to *Ensifer terangae*. In contrast, pSN0729-62 could be transferred to *Ensifer* but not to *Ochrobactrum*. These findings indicate that the host range of PromAγ and PromAδ plasmids may be different from that of PromAα and PromAβ plasmids, and the range may also be different between PromAγ and PromAδ plasmids. More in-depth comparisons on their host range should be performed to elucidate whether they have different ranges.

### The pSN1216-29 Plasmid Is a Member of a Previously Unrecognized Plasmid Group

The pSN1216-29 plasmid contains a *repA* gene showing high identity with pEC743_4 (37,000 bp) from *E. coli* (accession: CP015073), pKPN-704 (36,707 bp) from *K. pneumoniae* (Conlan et al., 2016), pSRC119-A/C (174,068 bp) from *Salmonella enterica* (Harmer et al., 2015), and pJHX613 (36,454 bp) from *P. aeruginosa* (Xiong et al., 2017) (Supplementary Table S5). It should be noted that these plasmids were all found in clinical isolates. The *repA* gene of these plasmids was clearly distinct from that of the other Inc groups including IncP-1, IncW, and PromA (Figure 1). Thus, these plasmids could be members of a new group of plasmids, and hereafter it is referred to as the pSN1216-29 family.

**FIGURE 3:**
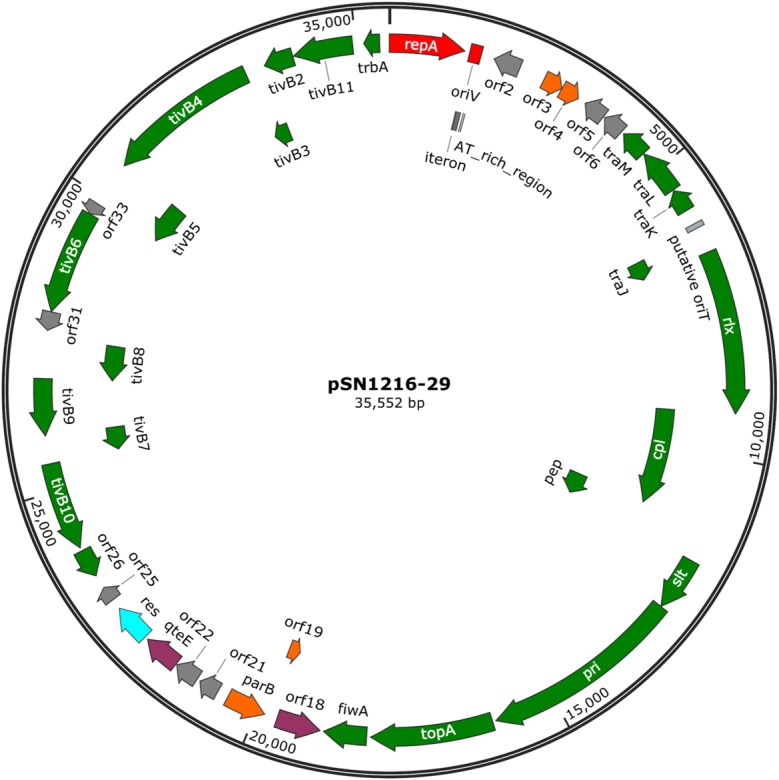
A circular map of plasmid pSN1216-29. Coding sequences are shown as arrows indicating their transcriptional direction. Colors indicate their putative functions: red, replication; orange, maintenance; green, conjugation; light blue, resolvase; gray, a hypothetical protein. The sequences of *oriV* and putative *oriT* are indicated by a rectangle.

**FIGURE 4:**
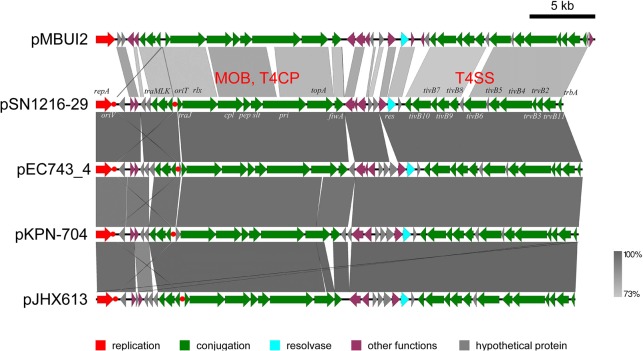
Alignment of pSN1216-29 with similar plasmids. Coding sequences of each plasmid are presented as colored arrows on the basis of their putative functions including MOB, T4CP, and T4SS for conjugation. Putative *oriV* and *oriT* sequences are indicated in red circles. Accession numbers of similar plasmids are given in Supplementary Table S5.

The pSN1216-29 plasmid contains a putative toxin–antitoxin system (orf3 and 4), a MOB_P_ (*rlx*) and MPF_T_ transfer system (*cpl, pep, slt, pri*, and *tivB2-11*), and other genes (Figure 3 and Supplementary Table S4-6). These turned out to be well conserved among pEC743_4, pKPN-704, and pJHX613 (Figure 4). It should be noted that putative genes involved in conjugation [for relaxase and type IV coupling protein (MOB, T4CP) and type IV secretion system (T4SS)] of pSN1216-29 share identity with those of pMBUI2 similarly isolated by the triparental exogenous plasmid capture method (Brown et al., 2013) (Figure 4). The order of CDSs is conserved between these two plasmids, and a putative nick site of *oriT* (5′-ATCTTG-3′) was found in the intergenic region between *traK* and *traJ* genes (Figure 3). Moreover, the *repA* gene of pSN1216-29 did not manifest high identity (<55% at the nucleotide sequence level, <10% at the amino acid sequence level). One study showed three specific features of pMBUI2: (i) a replication initiation gene (*rep*) different from that of the IncP-1 plasmid (*trfA*), (ii) a partitioning gene (*rep* upstream), and (iii) the absence of detectable iterons (Brown et al., 2013). These features were found to flank the *rep* gene of pMUBI2, and such a region was not found in pSN1216-29 or in the other three plasmids (Figure 4). The *rep* gene of pMBUI2 showed high identity with putative genes of plasmids in the draft sequences of *Enterobacteriaceae*, genera *Pseudomonas* and *Xanthomonas*.

Another interesting point regarding pSN1216-29 is that a part of the DNA regions (13.5 kb, including *repA*-*oriV* and genes for T4SS) share identity with those of pSRC119-A/C of *S. enterica* serovar Senftenberg strain SRC119 isolated from a pig (Harmer et al., 2015) (Supplementary Figure S5). This plasmid was identified as an IncC plasmid and was named as IncA/C_2_; however, IncA and IncC plasmids are compatible (Ambrose et al., 2018). Hereafter, we refer to this plasmid as an IncC plasmid, and it carries multidrug resistance genes including resistance to Km and neomycin (*aphA1*), to Tc (*tetA*[D]), erythromycin (*erm*[42]), apramycin, netilmicin, and tobramycin (*aacC4*), hygromycin (*hph*), sulfonamides (*sul1*), and spectinomycin and streptomycin (*aadA2*) (Harmer et al., 2015). The DNA regions containing *repA* and its surrounding region and genes for T4SS proteins (including genes *trbA* and *tivB2-11*) are highly conserved between pSN1216-29 and pSRC119-A/C (>95% nucleotide sequence identity; Supplementary Figure S5).

### The pSN1216-29 Plasmid Successfully Replicated in Different Bacterial Classes

**FIGURE 5:**
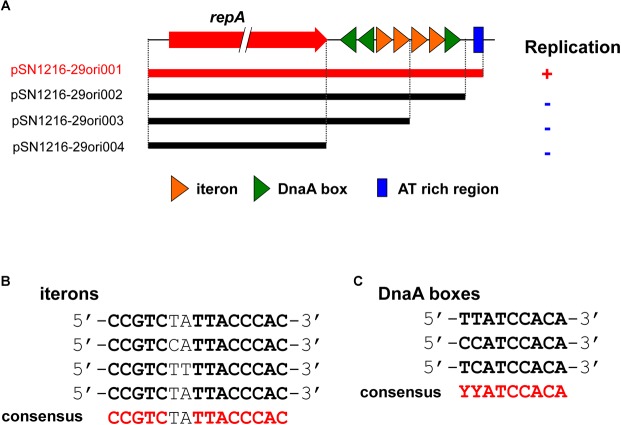
Structure of *repA* and the *oriV* region of pSN1216-29 used to construct the serial miniplasmids **(A)**. The red arrow indicates the *repA* gene, colored triangles show putative iterons (orange) and DnaA boxes with their direction. Capacity for replication of miniplasmids with the Tc^r^ gene in *Escherichia coli* JM109 is denoted by a plus (+) or minus (–) sign. Alignments of putative iterons **(B)** and *DnaA* boxes **(C)**.

In contrast to pMBUI2 (Brown et al., 2013), four tandem 15 bp repeat sequences and putative iterons were found downstream of the *repA* gene, [5′-CCGTC(T/C)(A/T)TTACCCAC-3′], and three putative DnaA boxes (Figure 5), with sequences (5′-YYCCACA-3′, Y indicates T or C) matched consensus sequences proposed previously (Schaefer and Messer, 1991). In addition, a 35 bp AT-rich region was found downstream (Figure 5A). These DNA regions were predicted to be an *oriV* of plasmid pSN1216-29, and then serial miniplasmids (pSN1216-29ori001 to pSN1216-29ori004) were constructed to confirm the minimum region for the replication of pSN1216-29. Transformants were successfully obtained with pSN1216-29ori001 containing *repA* and four putative iterons, three DnaA boxes, and an AT-rich region [transformation efficiency was at least 1–3 × 10^4^ colony-forming units (CFU) per pmol of plasmid DNA], whereas transformants were not yielded by the other miniplasmids [<1–2 × 10^2^ CFU/(pmol plasmid DNA); Figure 5A]. It was therefore concluded that pSN1216-29ori001 includes the minimum DNA regions for the replication of pSN1216-29. It should be noted that the nucleotide sequences of putative iterons, DnaA boxes, and the AT-rich region of *oriV* in pSN1216-29 are conserved in other pSN1216-29 family plasmids (pEC743_4, pKPN-704, and pJHX613), and pSRC119-A/C (data not shown).

The pSRC119-A/C plasmid contains another replication initiation gene (*repA/C*_2_) very similar to that in an IncC plasmid, and a large number of IncC plasmids were previously found in various Gram-negative bacteria, including genera *Klebsiella, Escherichia, Salmonella, Yersinia, Photobacterium, Vibrio*, and *Aeromonas* (Harmer and Hall, 2015). These hosts belong to one class: *Gammaproteobacteria*. Of note, pSN1216-29 was capable of transfer and replication not only in the *Gammaproteobacteria* strain (*E. coli, P. putida*, or *P. resinovorans*) but also in *Alphaproteobacteria* (*Ensifer terangae*) according to its filter mating assays. Therefore, the host range of an ancestral plasmid of pSRC119-A/C possibly has become broader via recruitment of DNA regions involved in the replication of pSN1216-29 family plasmids, although it was not transferred to *D. acidovorans* or *Hydrogenophaga* (*Betaproteobacteria*). This observation indicates that pSN1216-29 family plasmids are important for spreading antibiotic resistance genes; however, more in-depth analyses of their host range are required.

## Conclusion

In this study, we successfully isolated new broad-host-range plasmids from different environmental samples by an exogenous plasmid capture method. These plasmids were assumed to be cryptic and potential vehicles of various genes, including antibiotic resistance genes; therefore, it is important to monitor their distributions in nature. In addition, they may serve as new vectors for several bacterial strains compatible with other broad-host-range plasmids such as the pBBR1MCS series. The findings of the present study should facilitate the research on self-transmissible plasmids in environments and on the spread of antibiotic resistance genes. In-depth investigation of their abundance and host ranges in different environmental microbial communities will elucidate how they spread in natural environments.

## Author Contributions

MS conceived, designed, and supervised the study. KY, YM, HN, MT, and MS performed the experiments and analyzed the data. RM and HD designed and performed the experiments and the analyses of next-generation sequencing. MS, RM, HD, KI, MO, and KK wrote, reviewed, and edited the manuscript. All authors read and approved the final manuscript.

## Conflict of Interest Statement

The authors declare that the research was conducted in the absence of any commercial or financial relationships that could be construed as a potential conflict of interest.

## Footnotes

1 ftp://ftp.ncbi.nlm.nih.gov/genomes/GENOME_REPORTS/plasmids.txt

2 http://www.snapgene.com/
