# Supplementary material for: Novel Self-Transmissible and Broad-Host-Range Plasmids Exogenously Captured From Anaerobic Granules or Cow Manure
Source: Front Microbiol. 2018 Nov 6;9:2602. doi: 10.3389/fmicb.2018.02602 (PMC6232296; doi:10.3389/fmicb.2018.02602)
Supplement: Supplementary file 10 [file Data_Sheet_1.pdf]

Supplemental materials

Novel self-transmissible and broad-host-range plasmids exogenously captured from anaerobic granules or cow manure

Yanagiya, Maejima, Nakata et al.

Correspondence: shintani.masaki@shizuoka.ac.jp

The Supplementary Materials include:

- ✓ Supplemental Text S1
- ✓ Figures S1-S5
- ✓ Tables S1-S9 (provided in Excel format)
- ✓ References

Supplemental Text S1

Preparation of **Pseudomonas resinovorans** *CA10dm4RGFP*

The miniTn*7*(Gm)$P_{A1/O4/O3}$-*gfp*-a (Lambertsen et al., 2004)-harboring *E. coli* strain, and two other helper *E. coli* strains with pRK2013 (Figurski and Helinski, 1979) and pUX-BF13 (Lambertsen et al., 2004) were grown overnight in Luria broth (LB) with appropriate antibiotics at 37°C and 200 rpm, and a spontaneous Rif-resistant strain of *P. resinovorans* CA10dm4 (Shintani et al., 2006) was grown in LB with Rif at 30°C and 200 rpm. After harvest and wash the resultant cultures with LB, they were mixed and subjected to filter mating (30°C, overnight). Then, the mixture on the filter was resuspended with LB and then spread on a LB+Rif+Gm agar plate. After incubation on the selective plates for 2 d, colonies with GFP fluorescence were picked and isolated. The resultant strain was named *P. resinovorans* CA10dm4RGFP.

Filter mating assays with strains belonging to **Alpha- and Betaproteobacteria**

First, filter mating was performed between donor (*P. resinovorans* CA10dm4RGFP with each of pSN1104-11, pSN0729-62, and pSN1216-29) and recipient (*Pseudomonas putida* SMDBS) strains (Shintani et al., 2014). Km was used as a selective marker, carried by pBBR1MCS-2. PCR with *repA* primers for each plasmid and BOX-PCR were similarly performed as described in the main text to confirm the presence of the plasmid. The capability of the recipients' growth under anaerobic condition was assessed with LB containing KNO_3 in an anaerobic chamber (COY Laboratory Products) containing a N_2 atmosphere at 30°C. For the facultative anaerobes (*Ensifer*, *Ochrobactrum*, *Rhizobium*, and *Hydrogenophaga*), filter mating and spreading on the selective plates (LB with antibiotic [Gm] and KNO_3) were performed in the anaerobic chamber. Because *Delftia* is an obligate aerobe and has natural Km resistance, we prepared SMDBS strains with each plasmid and pBBR1MCS-5 (carrying Gm resistance gene) (Kovach et al., 1995).

Preparation of miniplasmids of **repA-oriV** *region of pSN1216-29*

Constructions of miniplasmids with *repA-oriV* region of pSN1216-29 was also performed with NEBuilder Assembly system (New England Biolabs), and primers were designed by NEBuilder v1.12.17. Amplification of a 1640-bp DNA region containing *repA* and *oriV* of pSN1216-29 (for pSN1216-29ori001, Table 1) was performed by PCR using with 29_repA_oriV_Fwd and 29_repA_oriV_Rev primers (Table S1), and that of Tc^r-gene was with 29_Tc_resistance_Fwd and 29_Tc_resistance_Rev (Table S1). Similarly, the 1590-bp (for pSN1216-29ori002), 1491-bp (for pSN1216-29ori003), and 1350-bp (for p SN1216-29ori004) DNA regions were amplified with 29_noAT_Fwd and 29_repA_oriV_Rev, 29_2iteron_Fwd and 29_repA_oriV_Rev, and 29_nooriV_Fwd and 29_nooriV_Rev (note that the same Rev primers were used for the construction of pSN1216-29ori001 to 003). As for the DNA fragment with Tc^r gene,

29_Tc_resistance_Fwd and 29_Tc_noAT_Rev (pSN1216-29ori002), 29_Tc_resistance_Fwd and 29_Tc_2iteron_Rev (pSN1216-29ori003), and 29_Tc_resistance_Fwd and 29_Tc_nooriV_Rev (pSN1216-29ori004) were used for PCR (note that the same Fwd primers were used for pSN1216-29ori001 to pSN1216-29 ori004). All of the PCR conditions with PrimeSTAR® GXL (TAKARA BIO) were as follows: 30 cycles of 98°C for 10 s, 55°C for 15 s, and 68°C for 2 min (pSN1216-29ori001); and 30 cycles of 98°C for 10 s, 55°C for 15 s, and 68°C for 1.5 min (pSN1216-29ori002-004).

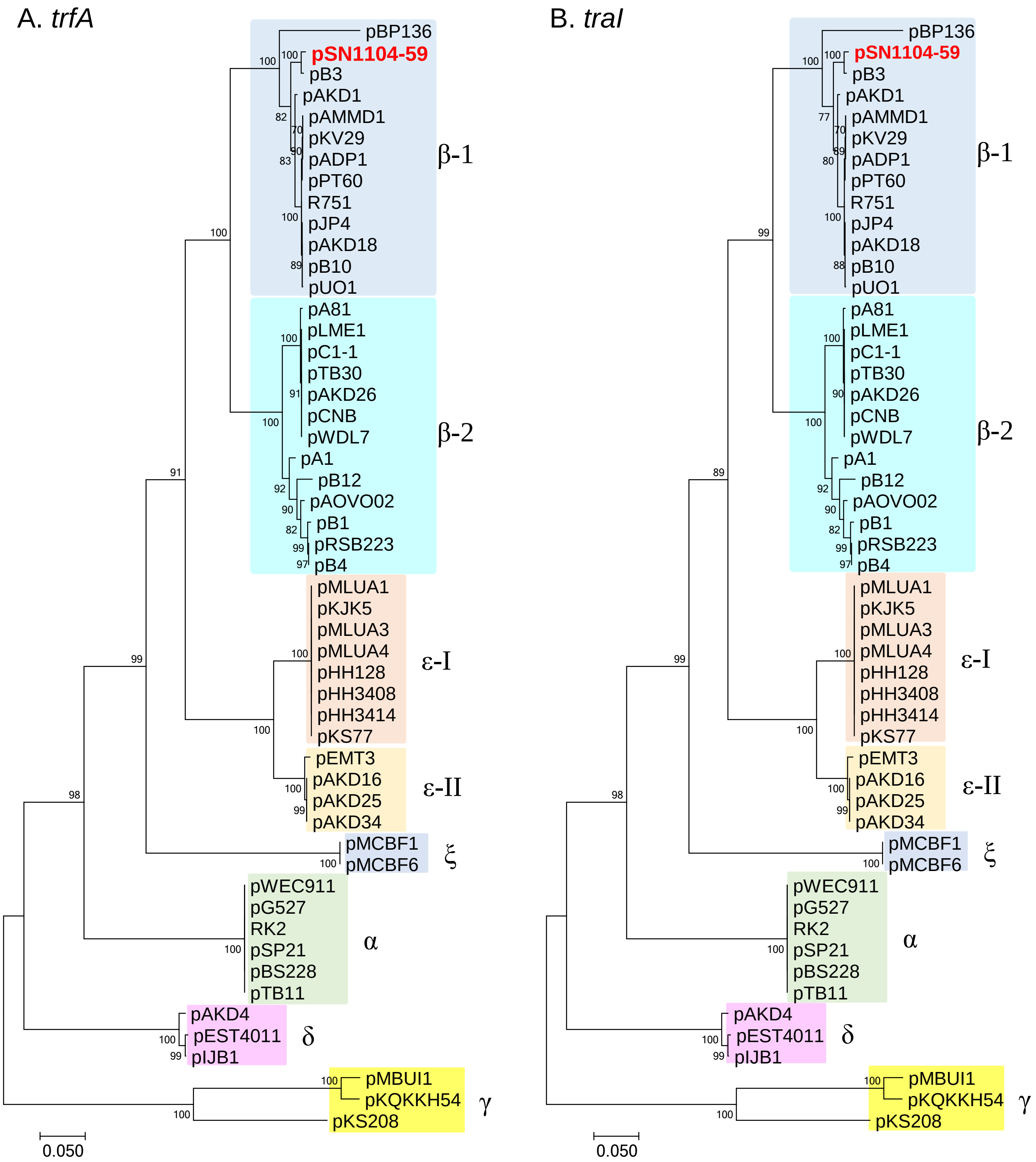

Figure S1. Phylogenetic analyses of IncP-1 plasmids with pSN1104-59. A phylogenetic tree was constructed from nucleotide sequences of *trfA* (A) and *traI* (B) by the maximum likelihood method with bootstrap percentages indicated at the nodes (Tamura-Nei model). A scale bar (0.050) shows the substitutions per nucleotide position. Previously proposed IncP-1 subgroups are highlighted in colors. The accession numbers of the reference plasmids are: pBP136(NC_008459), pB3(AJ639924), pAKD1(JN106164), pAMMD1(NC_008385), pKV29(JN648090), pADP1(NC_004956), pPT6(AM048832), R751(U67194), pJP4(AY365053), pAKD18(JN106169), pB10(AJ564903), pUO1(NC_005088), pA81(NC_006830), pLME1(NC_019263), pC1-1(HQ891317), pTB30(NC_016968), pAKD26(JN106171), pCNB(NC_010935), pWDL7(GQ495894), pA1(NC_007353), pB12(JX469826), pAOVO02(NC_008766), pB1(JX469829), pRSB223(JX469828), pB4(AJ431260), pMLUA1(KC964605), pKJK5(AM261282), pMLUA3(KC964606), pMLUA4(KC964607), pHH128(JQ004406), pHH3408(JQ004407), pHH3414(JQ004408), pKS77(JQ004409), pEMT3(JX469827), pAKD16(JN106167), pAKD25(JN106170), pAKD34(JN106175), pMCBF1(AY950444), pMCBF6(EF107516), pWEC911(JX469833), pG527(JX469830), RK2(BN000925), pSP21(CP002153), pBS228(NC_008357), pTB11(AJ744860), pAKD4(GQ983559), pEST4011(NC_005793), pIJB1(JX847411), pMBUI1(JQ432563), pKQKKH54(AM157767), and pKS208(JQ432564).

A

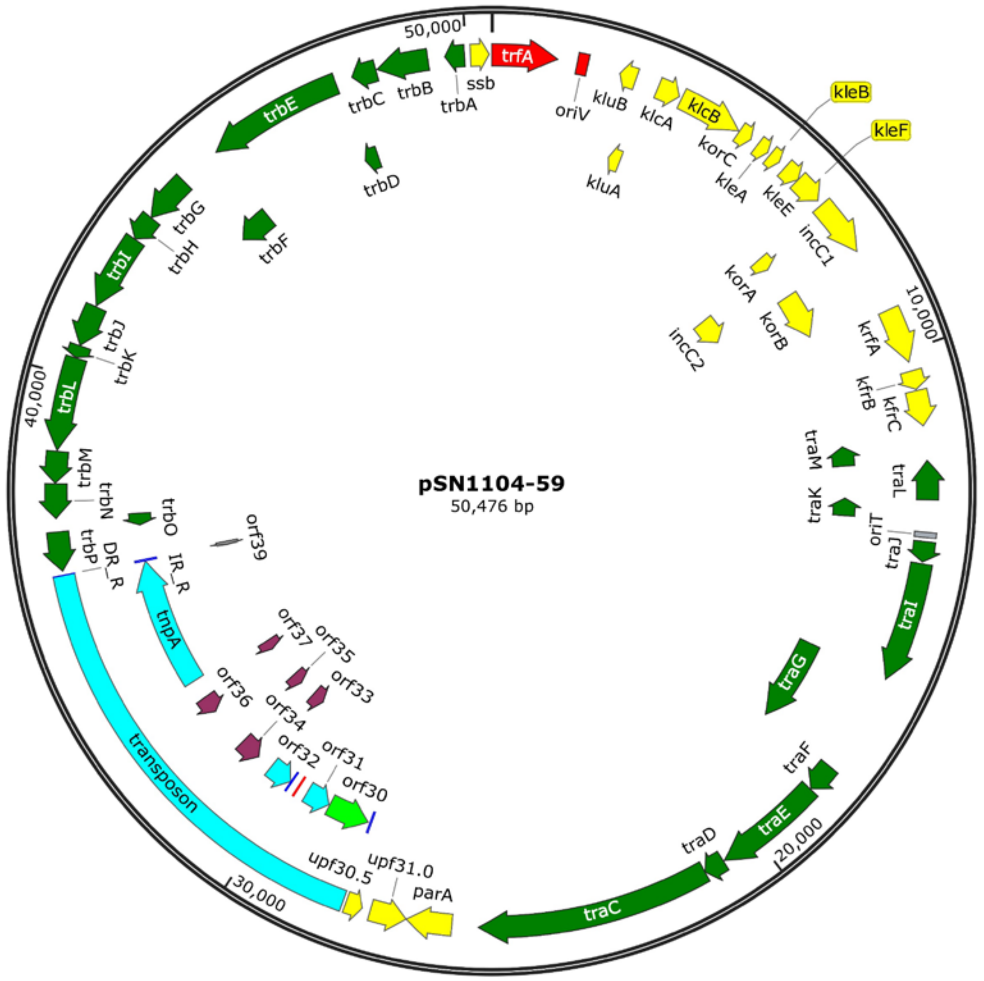

B

```
IR-L (→)  5′-GGGGTCGTCTCAGAAAACGGAAAAAATCGTACG-3′
IR-R (←)  5′-GGGGTCACCTCAGAAAACGGAAAAAATCGTACG-3′
             ******  *************************
```

Figure S2. (A) A circular map of plasmid pSN1104-59. Coding sequences are shown as arrows indicating their transcriptional direction. Colors indicate their putative functions: red, replication; orange, maintenance; green, conjugation; light blue, resolvase; gray, a hypothetical protein. The sequences of *oriV* and putative *oriT* are indicated by a rectangle. (B) Inverted repeat (IR) sequences found in both ends of a transposon containing 33-bp conserved nucleotides sequences (asterisks).

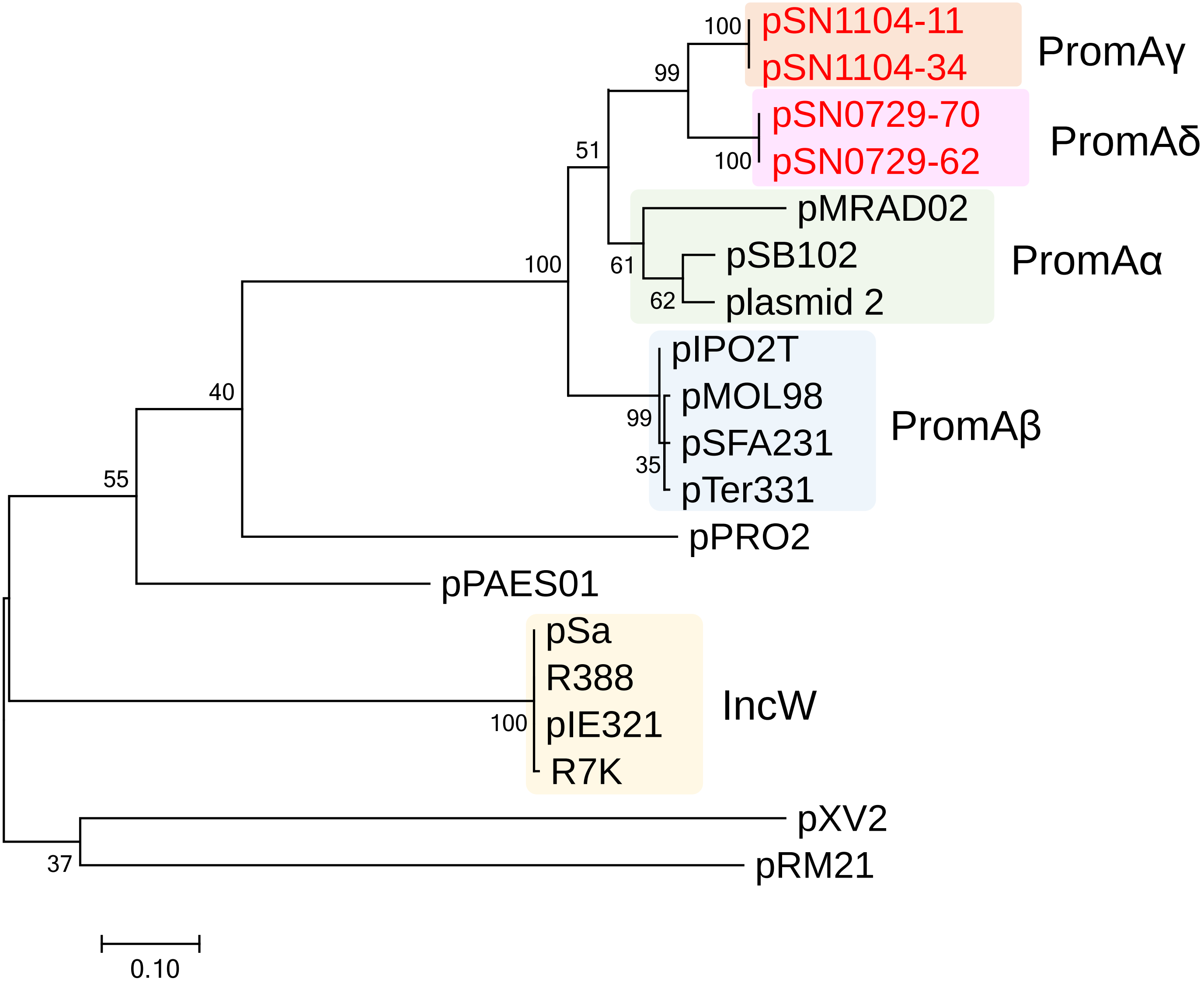

Figure S3. Phylogenetic analyses of PromA-like plasmids and IncW plasmids. Phylogenetic tree was constructed from amino acid sequences of replication initiation protein (RepA) by the maximum likelihood method with bootstrap percentages at the nodes (Tamura-Nei model). A scale bar (0.50) shows substitutions per amino acids position. IncW and subgroups of PromA group are highlighted in colors with the newly proposed subgroups PromAγ and PromAδ. The accession numbers of the reference plasmids are described in the legend of Figure 1.

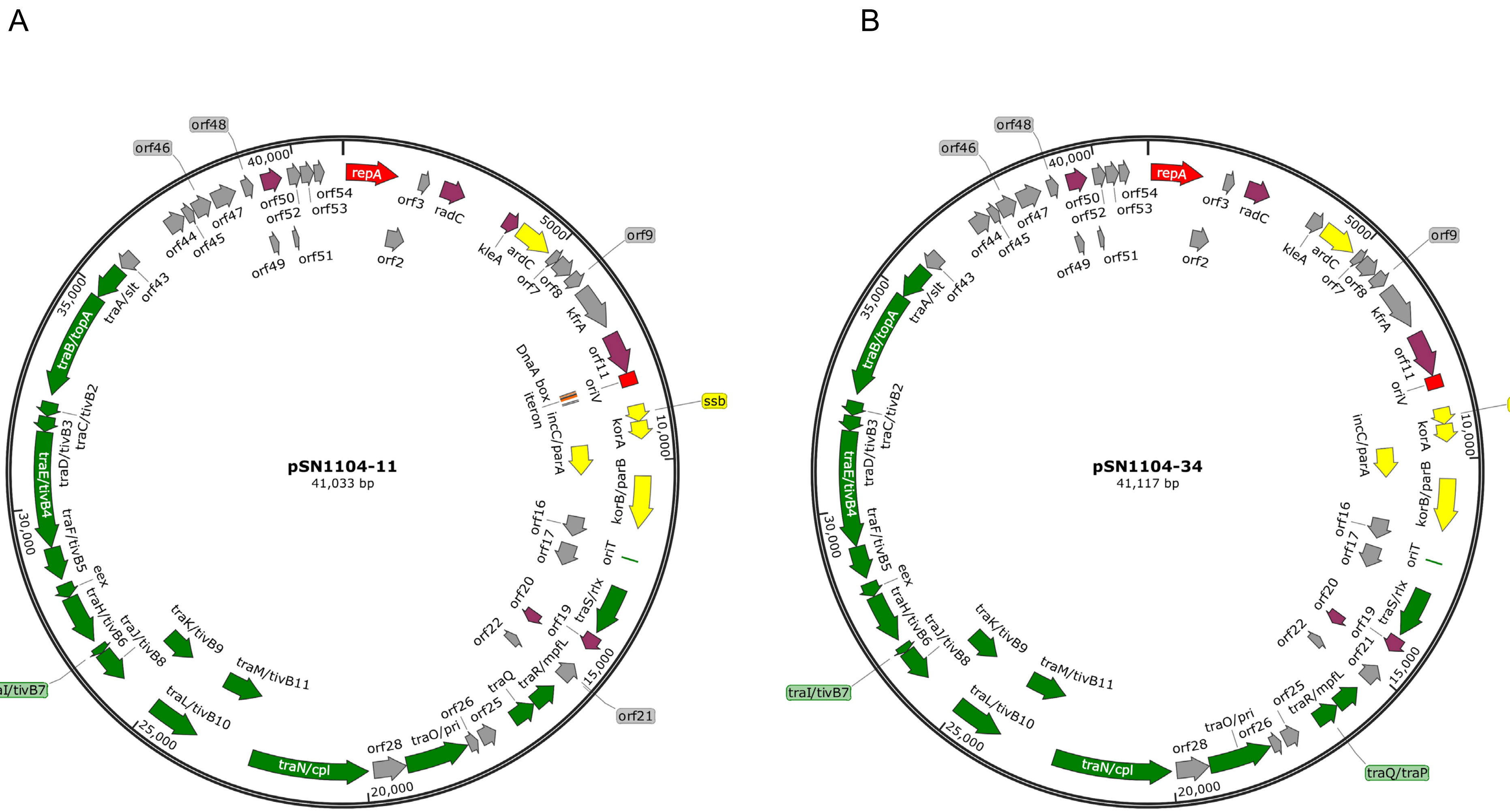

Figure S4. Circular maps of plasmids (A) pSN1104-11, (B) pSN1104-34, (C) pSN0729-62, and (D) pSN0729-70. CDSs are shown in arrows indicating their transcriptional direction. Colors indicate their putative functions: red, replication; orange, maintenance; green, conjugation; light blue, resolvase; gray, a hypothetical protein. The sequences of *oriV* and putative *oriT* are indicated by a rectangle.

C D

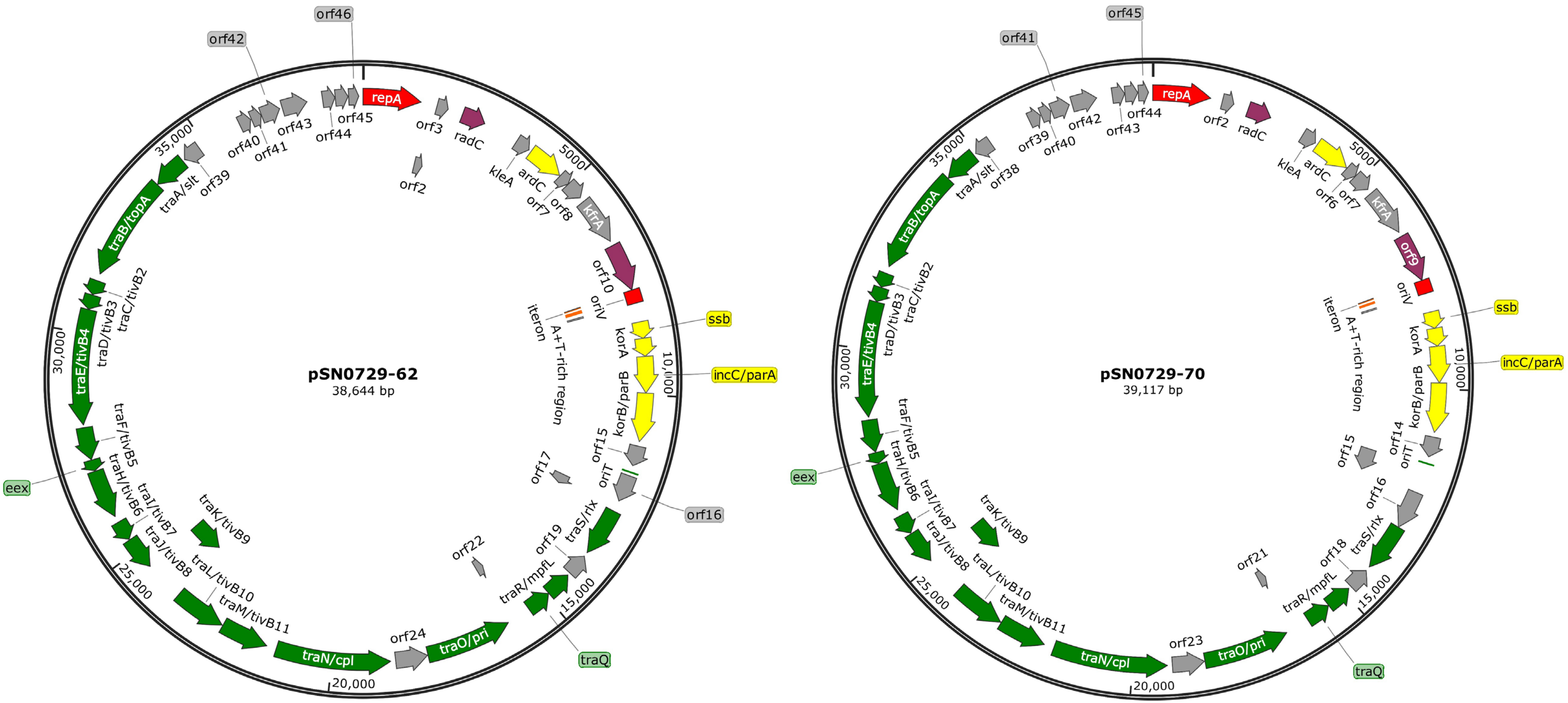

Figure S4. continued.

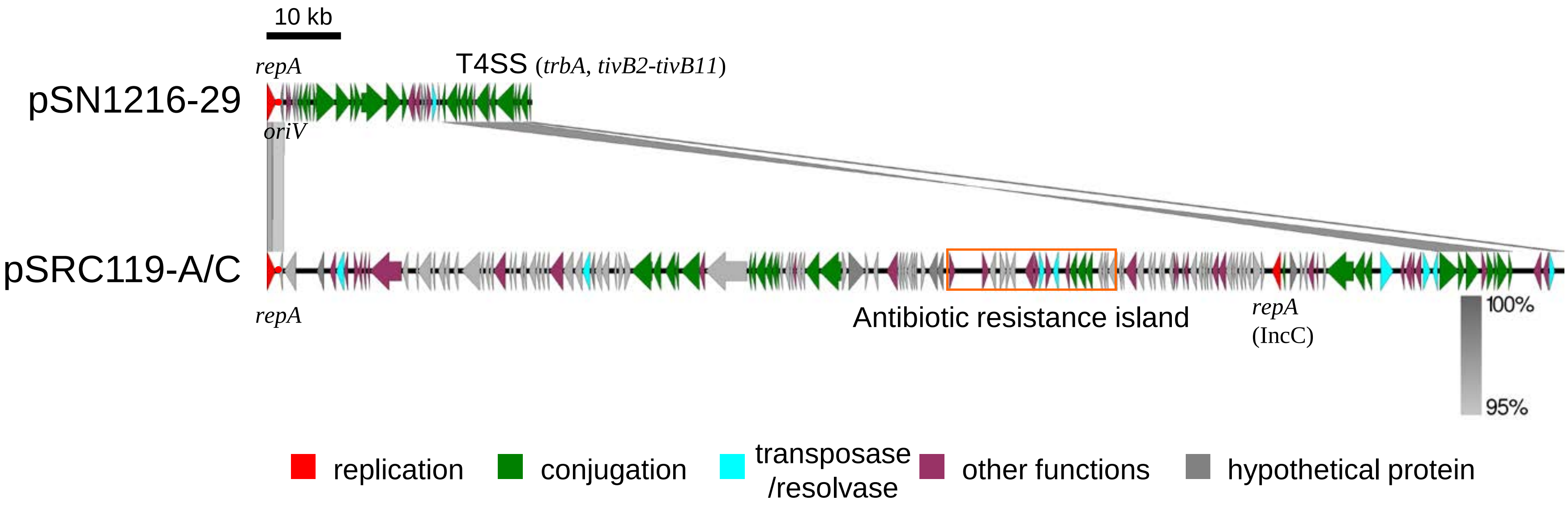

Figure S5. Alignment of pSN1216-29 with pSRC119-A/C found in *Salmonella enterica* serovar Senftenberg isolate SRC119 (Harmer et al., 2015). Coding sequences of each plasmid are presented as colored arrows on the basis of their putative functions.

References

Figurski, D.H., and Helinski, D.R. (1979). Replication of an origin-containing derivative of plasmid RK2 dependent on a plasmid function provided in trans. *Proc Natl Acad Sci U S A* 76**,** 1648-1652.

Harmer, C.J., Holt, K.E., and Hall, R.M. (2015). A type 2 A/C_2 plasmid carrying the *aacC4* apramycin resistance gene and the *erm*(42) erythromycin resistance gene recovered from two *Salmonella enterica* serovars. *J Antimicrob Chemother* 70**,** 1021-1025.

Kovach, M.E., Elzer, P.H., Hill, D.S., Robertson, G.T., Farris, M.A., Roop, R.M., and Peterson, K.M. (1995). Four new derivatives of the broad-host-range cloning vector pBBR1MCS, carrying different antibiotic-resistance cassettes. *Gene* 166**,** 175-176.

Lambertsen, L., Sternberg, C., and Molin, S. (2004). Mini-Tn*7* transposons for site-specific tagging of bacteria with fluorescent proteins. *Environ Microbiol* 6**,** 726-732.

Shintani, M., Matsui, K., Inoue, J., Hosoyama, A., Ohji, S., Yamazoe, A., Nojiri, H., Kimbara, K., and Ohkuma, M. (2014). Single-cell analyses revealed transfer ranges of IncP-1, IncP-7, and IncP-9 plasmids in a soil bacterial community. *Appl Environ Microbiol* 80**,** 138-145.

Shintani, M., Yano, H., Habe, H., Omori, T., Yamane, H., Tsuda, M., and Nojiri, H. (2006). Characterization of the replication, maintenance, and transfer features of the IncP-7 plasmid pCAR1, which carries genes involved in carbazole and dioxin degradation. *Appl Environ Microbiol* 72**,** 3206-3216.
